# Novel Orally Swallowable IntelliCap^®^ Device to Quantify Regional Drug Absorption in Human GI Tract Using Diltiazem as Model Drug

**DOI:** 10.1208/s12249-014-0172-1

**Published:** 2014-07-15

**Authors:** Dieter Becker, Jin Zhang, Tycho Heimbach, Robert C. Penland, Christoph Wanke, Jeff Shimizu, Kenneth Kulmatycki

**Affiliations:** 1 Vivo Drug Delivery GmbH, CH8832 Wollerau, Switzerland; 2 Drug Metabolism Pharmacokinetics Department, Novartis Pharmaceuticals Inc., East Hanover, New Jersey USA; 3 Novartis Pharma, Advanced Quantitative Sciences, Cambridge, Massachusetts USA; 4 Medimetrics Personalized Drug Delivery B.V., High Tech Campus 10-208, 5656 Eindhoven, Netherlands; 5 Medimetrics Personalized Drug Delivery Inc., Briarcliff Manor, New Jersey USA

**Keywords:** device, diltiazem, human study, intelliCap, regional absorption

## Abstract

Typically, colonic absorption of a drug is mandatory for a sustained release formulation to hold the drug’s plasma level for more than 12 or 24 h above the minimum therapeutic plasma concentration (efficacy). According to Drugs@FDA, only 7.4% of the oral drugs are extended release forms probably showing colonic absorption. Therefore an early determination of a drug’s colonic absorption using the IntelliCap® in animals or humans will provide the mandatory information to initiate or stop a SR form development. Diltiazem (60 mg) is used in the oral swallowable IntelliCap® and the marketed SR form from Mylan (coated beads). A human study with 14 healthy volunteers compared the Mylan formulation with the IntelliCap® device that releases the drug identical to the in-vitro dissolution of the Mylan product. The plasma profiles of IntelliCap® and Mylan formulation are highly similar. The mean AUC (bioequivalence fulfilled) and mean Cmax of IntelliCap® shows only a difference of +15% and −12%, respectively. But the PK profile of the Mylan formulation shows a broader peak around Cmax. About 81.8% diltiazem was absorbed in the colon (IntelliCap®) comparable to former publications. The Mylan is a SR diffusion coated beads form whereas the IntelliCap® is a monolithic capsule. The beads are transported in the gut and spread which results in a longer Tmax and a broader Cmax peak. The IntelliCap® device can quantitatively measure the colonic absorption of a drug in excellent accordance to a standard oral SR dosage form.

## INTRODUCTION

Many sustained release (SR) dosage forms are on the market. The main motivation for a new drug to develop a SR dosage form is a short half-live of the drug requiring two or more applications per day. Typically, a continuous absorption of the drug for 10–20 h is necessary to maintain plasma concentration of a drug to exceed the minimum therapeutic level.

The normal human transit time from ingestion of the dosage form to passing the illeo-ceacal valve is about 5 h (1) in fasted, healthy volunteers. If a drug has no colonic absorption and has a short half-life the transit time in the small intestine (<5 h) is typically not long enough to hold the drug’s plasma concentration for 12 or 24 h above the therapeutically necessary level. Therefore colonic absorption of a drug is mandatory to guarantee the drug’s efficacy and that bid or QD dosing can be applied.

According to the database Drugs@FDA (2), representing about 24,000 US drug products, only about 7.4% of all oral dosage forms (∼17,000) are extended release ones (1,200). The different number of dose strengths of one drug in a drug product can be neglected because the large number of products equals out this difference. Therefore one can conclude that also only 7.4% of all new drug substances will have properties that allow colonic drug absorption. That means for a new drug to be developed as SR form the chance for a successful market introduction is less than 7.4%. The FDA database is using the term extended release for all modified release forms such as delayed, sustained, pulsed release. Thus the sustained release dosage form part is assumed to be much lower. Therefore if a new drug requires a SR form an early determination of a drug’s colonic absorption is of high benefit but not routinely done in Pharmaceutical Industry.

A new oral swallowable device (IntelliCap®) now can be used in animals or humans (3) (4). Using the IntelliCap® system the information whether the drug does have sufficient colonic absorption can be provided quickly. Based on these facts an unambiguous decision to develop or stop the sustained release (SR) form can be made. This is beneficial for the Pharmaceutical Industry because there is a high likelihood that a new drug does not have colonic absorption and in this case the technical as well as clinical or pre-clinical development cost and time are lost. In addition, the capacity spent for such unsuccessful project cannot be used for a more promising one.

This paper is describing the first human study and its results using a drug (diltiazem) in the IntelliCap**®** to show how the regional absorption of a drug can be measured, quantified, and related to literature data.

## MATERIALS AND METHODS

### IntelliCap® Device

**Fig. 1:**
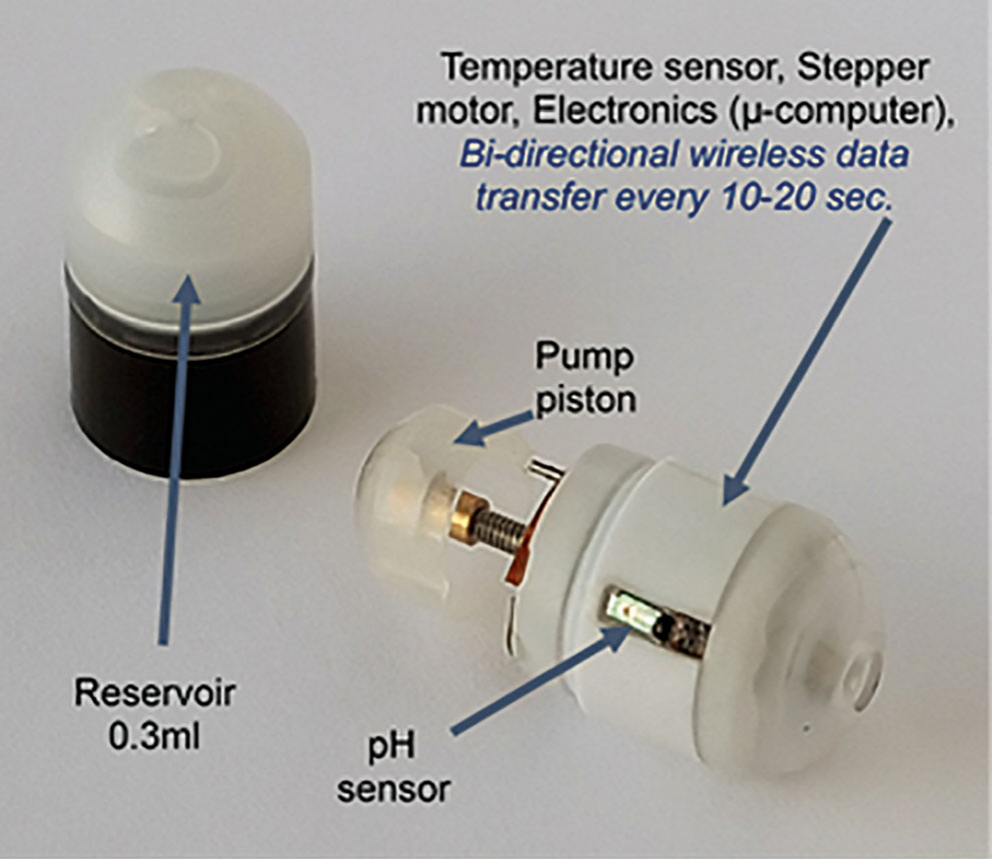
The IntelliCap® device and its parts

**Fig. 2:**
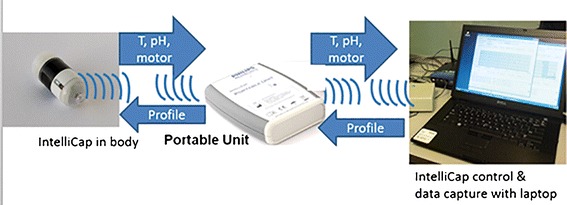
IntelliCap® wireless data exchange scheme

**Fig. 3:**
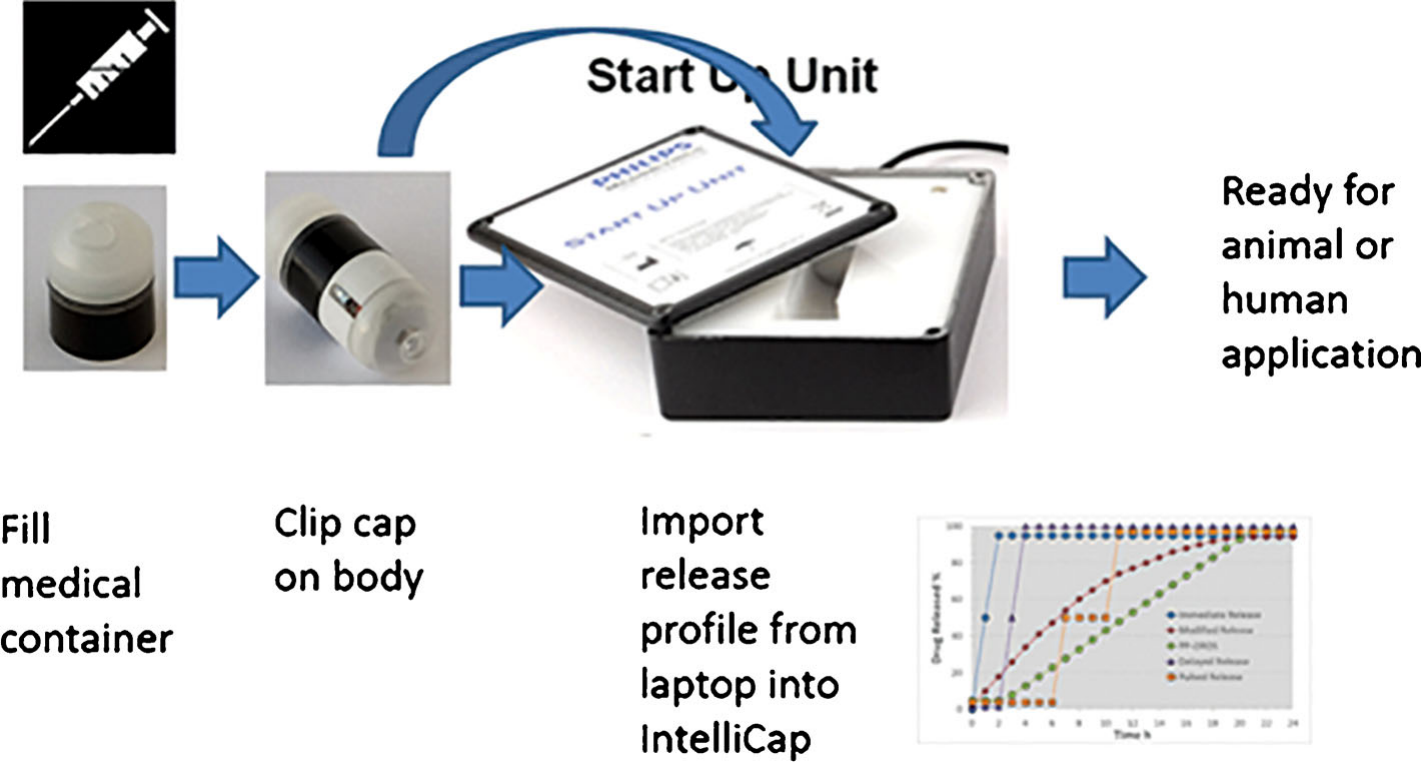
The set-up procedure for the IntelliCap®

The IntelliCap® device is an oral, single use, swallowable electronic device. It has the size of 000 hard-gelatin capsule (approximately 27 × 11 mm). It consists of a body and a cap. The cap is clipped onto the body. The cap consists of the medication container that can be filled with 0.3 ml of a drug liquid formulation (e.g., solution or suspension). The body contains a micro-computer and wireless data exchange unit, a temperature and pH sensor (accuracy ±0.1 pH units), a stepper motor with spindle and piston (see Figs. 1 and 2). The IntelliCap®’s μ-computer is programmed before ingestion to control the stepper motor and the release profile and location. By combining control of the stepper motor with variable time intervals allows to realize any drug release profile over time. In addition, the IntelliCap® can be also controlled manually in real-time from a laptop. For instance if a 6-h zero-order (linear) release profile has been programmed into the IntelliCap® the start of this profile can be commanded from the laptop when the IntelliCap® reached a particular location along the GI tract(e.g., the pylorus). Real-time localization of the IntelliCap® within the gut is achieved by monitoring individual pH profile (5). The set-up of the IntelliCap® before ingestion is described in Fig. 3.

### Test Article, Dose and Formulations

Diltiazem HCl (CID: 62920) extended release capsules 60 mg (Mylan Pharmaceuticals Inc., Morgantown, WV 26505, USA containing 55.1 mg diltiazem free base) was sourced by ProMedica Clinical Research Center (Brighton, MA). In this article, it is denoted Mylan ER® (extended release). Diltiazem-HCl powder was sourced as well by ProMedica Clinical Research Center (Brighton, MA). Diltiazem has a molecular weight of 414.5 (free form), a pKa of 8.06 (weak base), a water solubility of 465 mg/mL (6) and a log P of 2.79. Diltiazem is a BCS class 1 drug (7), bioavailability is about 40–50%, the drug is a substrate of CYP3A4 (N-demethylation, about 80%), of CYP2D6 (O-demethylation, about 10%), of esterases (desacetylation, about 10%) (8) and substrate of P-glycoprotein (9).

**Fig. 4:**
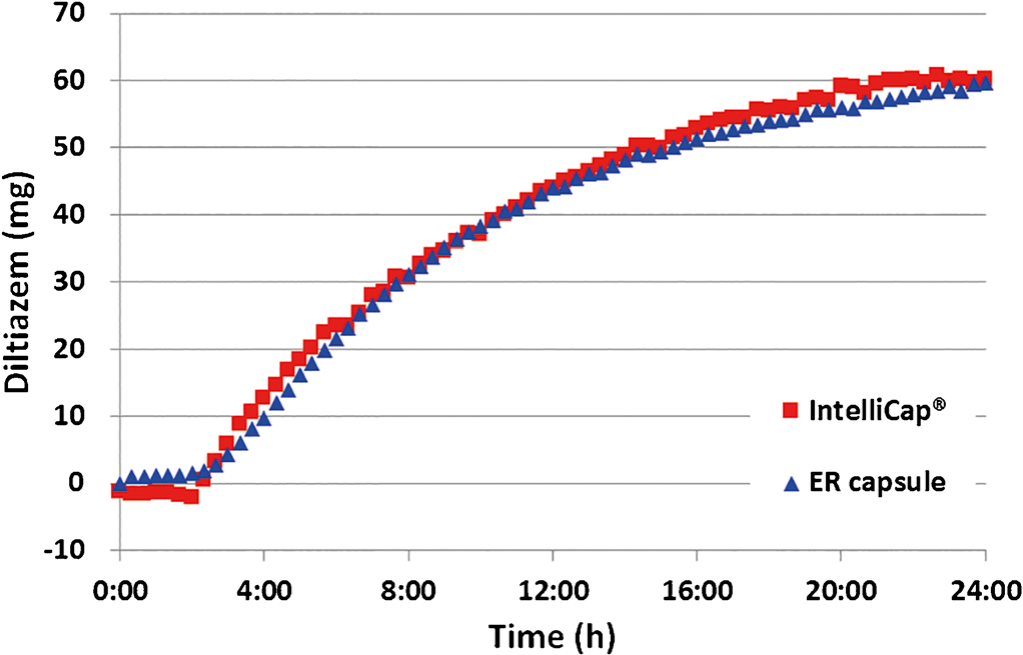
*In vitro* dissolution profile of Mylan ER® (60-mg capsule) and IntelliCap® releasing diltiazem solution (900 mL H_2_O, 37°C, 100 rpm, paddle). Diltiazem HCl was quantified spectro-photometrically at 239 nm

The IntelliCap® is filled with approximately 300 μL of an aqueous solution of diltiazem HCl (270 mg/mL). The IntelliCap® capsule is programmed to release approximately 220 μL of this solution over a time period of 24 h with a first-order release profile mimicking the release profile of the marketed 60-mg extended release capsule according to USP Drug Release Test 2 (Fig. 4).

The actual dose released during the gut transit from the IntelliCap® was determined by analyzing the remaining diltiazem content in the excreted IntelliCap®. The drug content was determined using a UV method. This drug amount was subtracted from the amount that was filled in the medication container. This procedure was done for all subjects except one that could not recover the IntelliCap® after excretion. In this subject, an abdominal X-ray confirmed (6 days after administration) that the capsule was not withhold. This results in a mean released dose of 64.8 mg.

### Clinical Study

The study used an open-label, randomized, two-period crossover design with a minimum washout period of 1 week. The study was approved by an Institutional Review Board (New England IRB, Newton, MA, USA) and was conducted at Promedica Clinical Research Center (Brighton, MA, USA). The human study is registered as ISRCTN45336965 (10).

### Health Volunteers

In total, 14 healthy, male, non-smoking volunteers were enrolled. To participate, subjects had to be between 18 and 45 years of age with a BMI between 18 to 29 kg/m^2^ and a minimum weight of 50 kg. Prior to administration, subject’s health status was examined including medical history, physical examination, vital signs, electrocardiogram, and laboratory tests (hematology, blood chemistry, urinalysis, hepatitis B and C, HIV). Subjects with swallowing disorders, implanted medical devices, gastrointestinal diseases (inflammatory bowel disease, ulcers, gastrointestinal or rectal bleeding), major gastrointestinal surgery were excluded from participation. Subjects taking prescription drug prior 1 month or over the counter medication within 2 weeks before administration were not enrolled into the study. Written informed consent was obtained from all participating volunteers.

### Dosing

After an overnight fast, subjects were administered at around 8 AM either one IntelliCap® capsule with diltiazem HCl aqueous solution (∼0.3 ml of 270 mg/mL) or one diltiazem extended release capsule (60 mg) with 240 mL of water. The IntelliCap® capsule was programmed to release its payload over time resembling the *in vitro* dissolution profile of the 60-mg Mylan ER capsule (Fig. 4) starting immediately after administration. Subjects remained fasted for 5 h after administration. After 5 h, subjects returned to a regular meal schedule.

### Sampling

Blood samples (6 mL) were collected at pre-dose, 1, 2, 3, 4, 5, 6, 7, 8, 9, 10, 11, 12, 16, 24, 36, and 48 h post-administration. Diltiazem was quantified in plasma using a validated liquid chromatography tandem mass spectrometry method with a lower limit of quantification of 0.2 ng/mL in 200 μL plasma. Human heparinized plasma samples (200 μl), 20 μl of D_4_-diltiazem internal standard and 800 μl acetonitrile are mixed and centrifuged. A portion of the supernant is injected in UPLC ESI+MS/MS. Quantitation of diltiazem is performed by using an analyte to internal standard ratio and a concentration-weighted linear regression.

### Determination of Gastrointestinal Transit Times

**Fig. 5:**
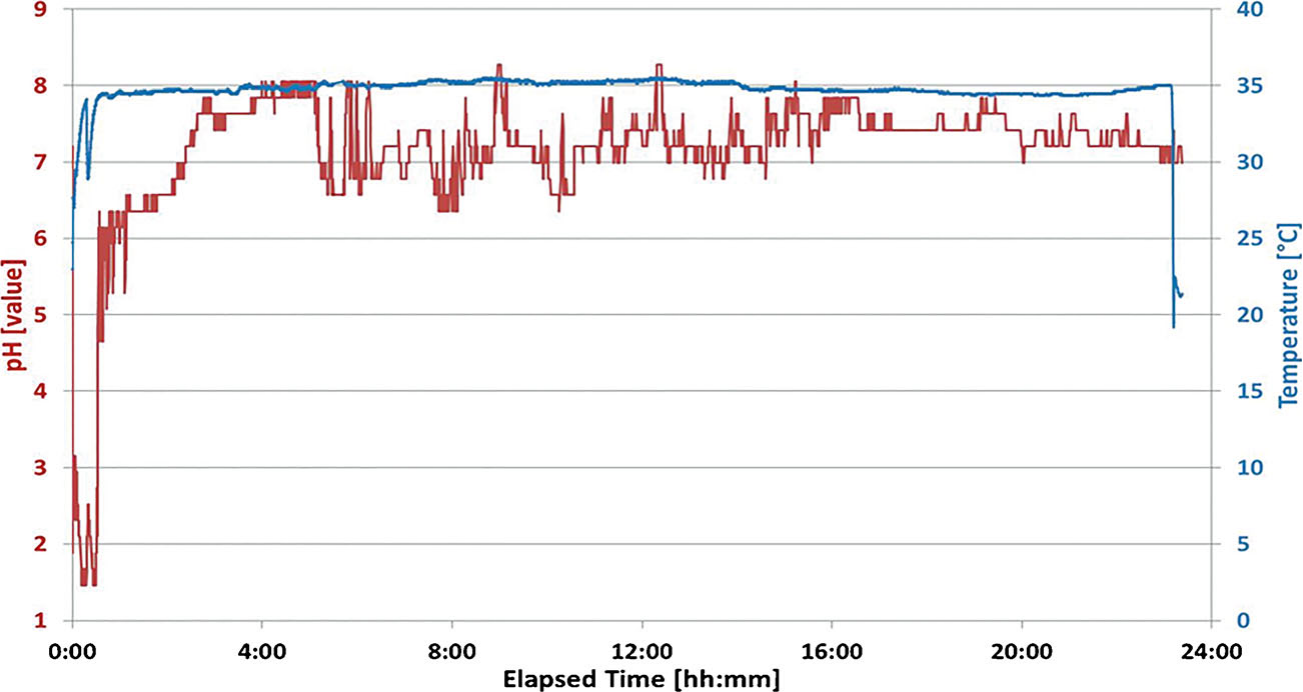
Localization of gut regions by pH and temperature data

Individual gastrointestinal transit times (gastric residence, small bowel transit, colon transit, and whole gut transit) were determined from subjects in the IntelliCap® arms using the pH and temperature/time data. The gastrointestinal pH and temperature profiles recorded by the IntelliCap® capsule were analyzed for capsule ingestion resulting in a rapid and sustained rise in temperature from room to body temperature. The pylorus passage is characterized by a rapid and sustained rise from the acidic stomach environment into the neutral pH of the duodenum (approximately pH 6). The ileo-cecal valve passage is observed when drop of >0.5 pH units occurs that is caused by the acidic bacterial digestion products in the colon. Finally, anal excretion causes a rapid and sustained drop in temperature from body to room temperature (see Fig. 5).

### Pharmacokinetics

The following plasma pharmacokinetic parameters have been determined for diltiazem:

$$ {\mathrm{AUC}}_{\mathrm{last}},{\mathrm{AUC}}_{0- t},{\mathrm{AUC}}_{\infty },{C}_{\max },{T}_{\max },{T}_{\mathrm{lag}},{T}_{1/2,}\mathrm{Vd}/ F,\mathrm{CL}/ F. $$

Pharmacokinetic parameters were determined using non-compartmental method(s) using WinNonlin Pro 6.2 (Pharsight, USA).

### Calculation of Regional Absorption

Gastro plus® version 8 (Simulations plus Inc.; Lancaster; USA) has been used to calculate regional absorption of this study (mean Cp *versus* time) by using a model having a goodness of fit *R* = 0.72. The iv data used in this model are taken from (11). The important parameters for the model for Diltiazem in the IntelliCap® are summarized in Table I

**Table I:** Important Parameters used in Gastro Plus to Model the Cp/Time Curve

Parameter	Value
Dose form	CR integral tablet
Solubility	465 mg/mL (pH 7.2)
logP neutral	2.79
Liver first pass	70%
Clearance	39.4 L/h

**Fig. 6:**
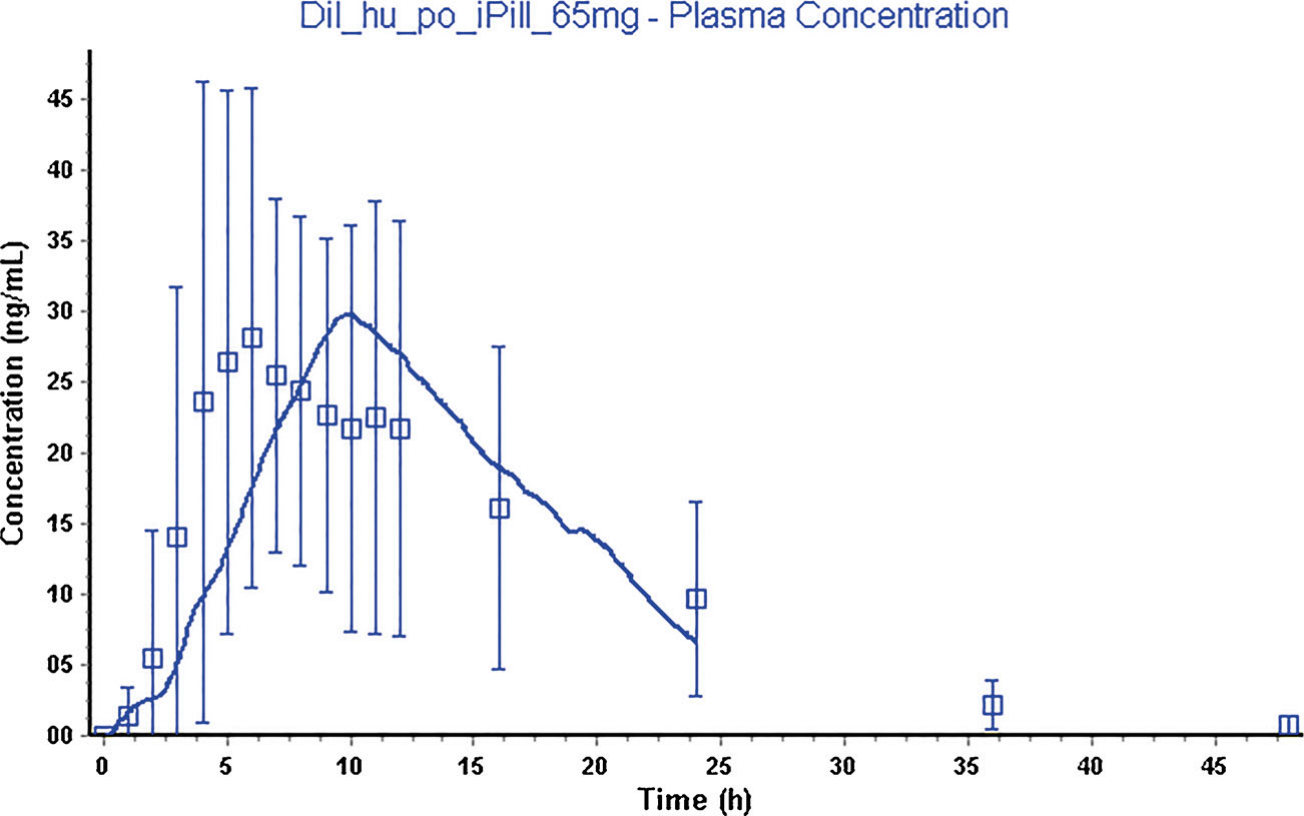
IntelliCap® mean drug plasma concentration *versus* time with standard deviation as error bars (*squares*: observed, *solid line*: simulated results from Gastro plus)

**Fig. 7:**
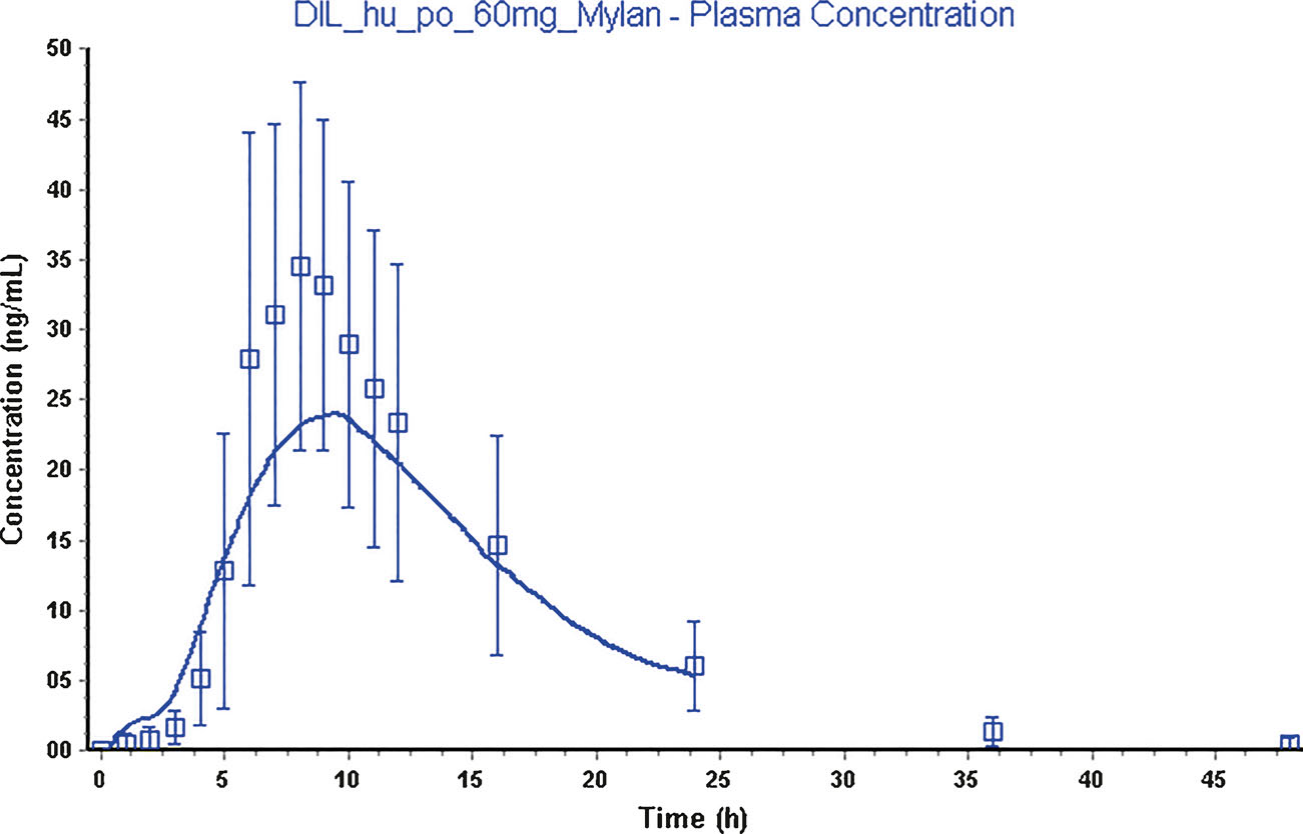
Mylan ER mean drug plasma concentration *versus* time with standard deviation as error bars (*squares*: observed, *solid line*: simulated results from Gastro plus)

The same model was used for the Mylan ER formulation as dose form “CR Dispersed” in Gastro Plus and had a goodness of fit of 0.75. The transit times of this formulation are not measured and therefore the IntelliCap® transit times were used. The observed plasma concentrations of diltiazem *versus* the simulated concentrations are shown in Figs. 6 and 7.

## RESULTS

All subjects enrolled in the study completed the study. The IntelliCap® system was safe and well-tolerated in healthy volunteers. No adverse events or adverse device effects were observed.

**Fig. 8:**
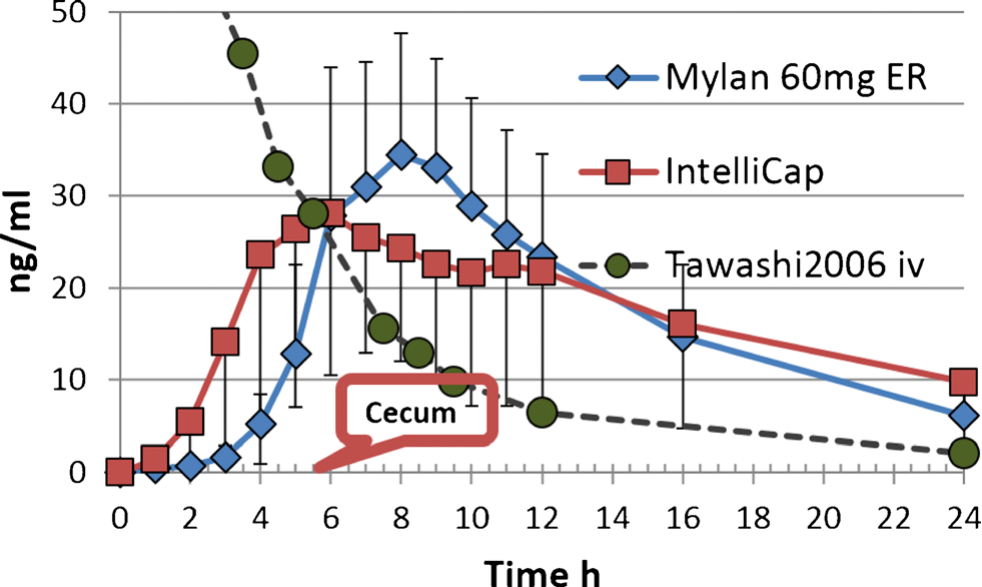
Plasma concentration *versus* time profile of the Mylan ER (diamonds) and IntelliCap® (*squares*) formulation in healthy volunteers (*N* = 14) and the iv bolus solution 20 mg (*circles*) from Reference (11). The cecum transit time of the IntelliCap is marked

PK data from all subjects were included in the statistical analysis. Summary plasma time profiles and statistics for the PK parameters are provided in Fig. 8 and Table II, respectively, and are also shown in (12).

**Table II:** Important PK Results % CV in Parenthesis (All Absorption Data Calculated with Gastro Plus)

PK parameter	Mean	Mean
Dosage form	IntelliCap®	Mylan ER
Dose (mg; diltiazem HCl)	64.8	60.0
Cmax (ng/ml)	34.7 (28.8)	39.5 (33.9)
AUClast (ng × h/ml)	494.0 (63.4)	428.0 (39.1)
Tmax (h)	6.3 (37.1)	8.0 (24.0)
Tlag (h)	0.5	0.7
*T*½ (h)	6.6 (22.3)	6.1 (20.0)
Volume of distribution/*F* (L)	1,600 (59.5)	1,420 (47.8)
Total clearance/*F* (L/h)	177 (65.1)	163 (47.1)
Absorption small intestine (%)	10.6	12.7
Absorption colon (%)	81.8	65.6
Total absorption (%)	92.4	78.3
Literature colonic absorption (%) (7)	82	

The mean cecum arrival time for the IntelliCap® is 5:34 hh:min (Table III). The Tmax for the IntelliCap® is 6:18 hh:min (Table II). There is a data caption rate of less than 1 min for the pH that determines the cecum arrival time compared to an hourly blood sampling. Considering these facts the Tmax and cecum arrival can be regarded nearly identical.

**Table III:** Mean Gastrointestinal Transit Times in IntelliCap® Arm (hh:mm)

	GRT	SBTT	CTT	WGTT
Mean	01:09	04:25	34:17	39:51
SD	01:04	01:03	27:00	27:21
Min	00:12	02:18	05:30	08:06
Max	03:44	06:19	98:47	104:05

*GRT* gastric residence time, *SBTT* small bowl transit time, *CTT* colon transit time, *WGTT* whole gut transit time

CTT and WGTT: in two subjects portable unit run out of battery power, determined from subject reported time of excretion

## DISCUSSION

The mean PK data of both formulations are very similar. Considering the Mylan ER formulation as reference the IntelliCap® deviates only slightly from the reference and within the error range especially for AUC +15% and Cmax −12%. But despite the fact that the dissolution profiles of both dosage forms superimpose, the PK profiles show differences:

- longer Tmax for Mylan ER
- higher Cmax for Mylan ER
- longer Tlag for Mylan ER
- less of the dose is absorbed by Mylan ER

The longer Tmax and higher Cmax of the Mylan formulation can be explained. The used dosage forms are different. The IntelliCap® is a monolithic unit and the Mylan ER are diffusion coated beads in a capsule. For multi-particulate systems it is well-known that they accumulate and stagnate in front of ileo-cecal valve (13–15). In addition the transit time in the small intestine is nearly invariable for monolithic or multiparticulate dosage forms (16) and also no difference can be seen in gastric emptying (17–19) for both forms. Therefore the IntelliCap® will arrive at the ileo-cecal valve approximately at the same time as the first pellets. The pellets tend to spread in the small intestine and will be transported when they arrive into the colon (20). This results in a later Tmax by about 2 h for the multiparticulate form and therefore they stay longer in the small intestine at least in part and increase Cmax. Consequently, at this time when the pellets are partly in the ileum and in the colon, the diltiazem plasma concentration profile is less steep after 6 h compared to the graph between 4 and 6 h.

The longer lag time seen in the mean plasma profiles is misleading probably because of the high variability. The calculated Tlag times are very similar (Table II).

**Fig. 9:**
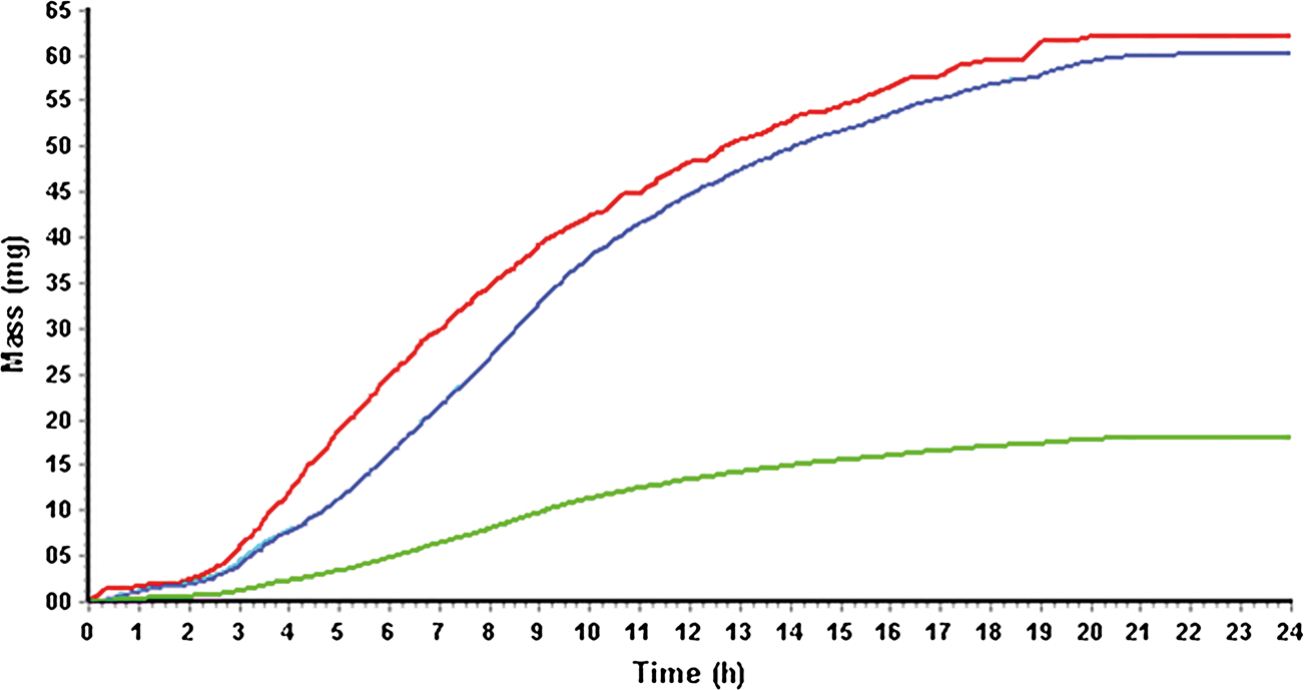
IntelliCap® results from Gastro plus: *In vivo* dissolution (*red solid line*), fraction absorbed Fa (*blue line*), and total systemic concentration (*green line*)

**Fig. 10:**
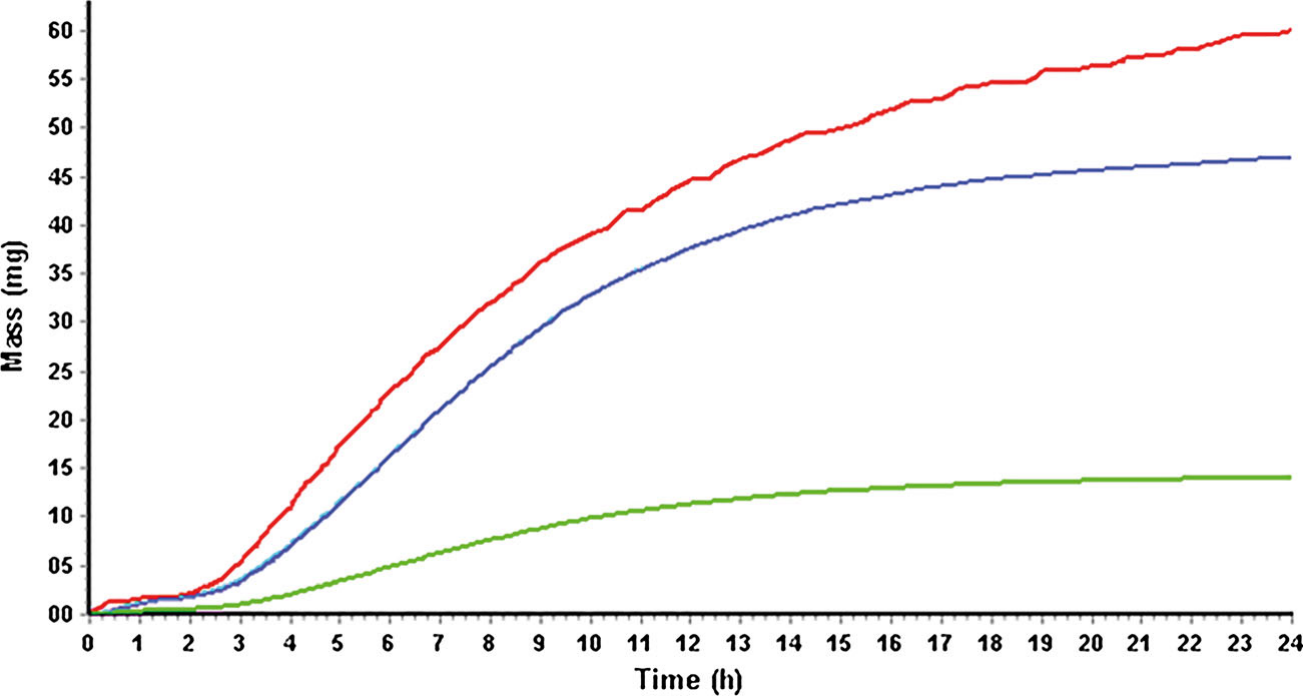
Mylan ER results from Gastro plus: *In vivo* dissolution (*red solid line*), fraction absorbed Fa (*blue line*) and total systemic concentration (*green line*)

The lower drug exposure produced by the Mylan ER is probably due to the water dependent passive diffusion release mechanism of the pellets. The IntelliCap® is using an active pump system that is releasing drug independently from the environmental water concentration especially in the colon. Therefore, due to the fact that water reduction in the colon is drastic (21) and water is the driving force for the drug release from the pellets. This is evident from Figs. 9 and 10 when comparing the *in vivo* dissolution and fraction absorbed showing a lower total Fa for the Mylan ER and a much stronger decline in Fa in the colon (between 12 and 24 h) compared to the IntelliCap®. This is also confirmed in an article by Lennernäs (7). There are two drugs among 42 drugs that have been used in two different formulations in the colon. AZ6 was intubated as solution and granules and dexloxiglumide was released from an Enterion capsule as solution and powder. In both cases the solution was better absorbed by the colon. This is in line with the observation that the active pump system of the IntelliCap in colon added about 0.2 ml to the overall colonic fluid volume of 10–20 ml (21). The Lennernäs (7) paper also stated that drugs showing high permeability do have better colonic absorption of about 70% relative to the absorption in the small intestine (100%) in comparison with low permeable drugs. But this analysis is based on drugs that all have colonic absorption which is a rare drug property.

Another indication of colonic absorption can be observed in the mean diltiazem plasma profiles. Typically for a drug that does not show any colonic absorption, the plasma profile will decline after reaching Cmax in a more or less exponential way comparable in shape to an iv injection as can be seen on Fig. 8 for diltiazem iv. If a drug like diltiazem has significant colonic absorption, the shape of the plasma profile after Cmax becomes different by the additional drug input as can be seen on Fig. 8 especially for the IntelliCap® profile between 6 and 12 h. According to Wilson (22), the transit time in the ascending colon is about 3 to 5 h and in the transverse colon 0.2 to 4 h. The colon entry time of the IntelliCap® is about 5.5 h and can be projected to be around 8.2 to 14.5 h entering the descending colon. This is in good accordance to the drug input seen in the colon between 6 and 12 h.

In addition, the plasma profile is showing a reduced input rate of diltiazem from the IntelliCap® between 6 and 12 h. This is also reported by Lennernäs (7) for diltiazem which had an increased Tmax and decreased Cmax when directly instilled into the colon compared to the absorption in the small intestine.

Interestingly, these differences observed of a drug released by an active pump *versus* passive diffusion in the pellets can be simulated using PBPK software as shown above. The device in combination with PBPK software can therefore contribute significantly in the elucidation of the quantitative relation of a drug’s local gut solubility, precipitation, metabolism in gut wall and the liver, and the impact of transporters resulting in the finally measured systemic drug plasma concentration profile. Especially because solubility, metabolism, and transporter impact can be measured *in vitro* and entered into PBPK models that predicts well the measured data. This will allow to simulate the currently often unknown part of precipitation of a drug in the gut by using the accurately known dose pumped out by the device. But this important aspect needs further investigations also because the regulatory agencies meanwhile show interest in PBPK simulation and work on guidelines (23).

## CONCLUSION

IntelliCap® determines and quantifies colonic absorption of a drug (using simulation software, e.g., Gastro plus) in excellent accordance to published data.

The study proves that the IntelliCap® is a very beneficial tool to assess the extent of colonic absorption for drug and thereby providing the mandatory information to make a scientifically sound early GO / NOGO decision on development of a modified release dosage form. This is of paramount importance for the Pharmaceutical Industry because a significant proportion of new drugs (>90%) do not have sufficient colonic absorption that would allow a sustained release formulation to be developed.
